# Microplastics in fecal samples of whale sharks (*Rhincodon typus*) and from surface water in the Philippines

**DOI:** 10.1186/s43591-021-00017-9

**Published:** 2021-09-26

**Authors:** Mila Mi Hua Yong, Clara Leistenschneider, Joni Anne Miranda, Maria Kristina Paler, Christine Legaspi, Elitza Germanov, Gonzalo Araujo, Patricia Burkhardt-Holm, Gabriel Erni-Cassola

**Affiliations:** 1 Man-Society-Environment (Programme MGU), Department of Environmental Sciences), University of Basel, Vesalgasse 1, CH-4051 Basel, Switzerland; 2 Large Marine Vertebrates Research Institute Philippines, 6308 Jagna, Bohol Philippines; 3 Department of Biology, University of San Carlos, Talamban, Cebu City, Philippines; 4 Marine Megafauna Foundation, 11260 Donner Pass Road, Unit 256, Truckee, CA 96161 USA; 5 Marine Research and Conservation Foundation, Somerset, TA4 3SJ UK

**Keywords:** Microplastic, Whale shark, Southeast Asia

## Abstract

Marine plastic abundance has increased over the past 60 years and microplastics (< 5 mm) constitute a primary component of such litter. Filter-feeding megafauna, such as the whale shark, might be particularly affected by microplastic pollution as their feeding mode requires filtration of up to thousands of cubic meters of water. In addition, the habitat range of whale sharks intersects with several recognized microplastic pollution hotspots, among which is the Coral Triangle. Direct evidence for microplastic ingestion in whale sharks however, has not yet been presented. Here we show that whale shark scat collected in the Philippines from 2012 to 2019 contained a mean of 2.8 microplastics g^− 1^. Contrary to our expectations, the microplastic concentration in the scat remained consistent from 2012 to 2019. Water samples from the study site in 2019 indicated that the local microplastic pollution (5.83 particles m^− 3^) was higher than in surface waters in other whale shark habitats, but well below other pollution hot-spots found in Southeast Asia and China (range: 100–4100 particles m^− 3^). With the predicted growth in plastic use, leading to increased plastic marine pollution, whale sharks are expected to become more exposed to this form of pollution. To what extent microplastic ingestion impacts the overall health status of this endangered species remains an open question.

**Supplementary Information:**

The online version contains supplementary material available at 10.1186/s43591-021-00017-9.

## Introduction

Marine plastic pollution is a global problem acknowledged under the United Nations Sustainable Growth Development Goal 14 - life below water. Improper waste management constitutes a primary source of marine plastic waste with an estimated yearly global input of approximately five to thirteen million metric tons of plastic material to the oceans [1]. Exposed to the environment, plastics break down into increasingly smaller particles [2], such as microplastics, which are most commonly considered as particles < 5 mm [3]. Given the demonstrated increase of marine plastic abundance over the past 60 years [4], and the well-established global distribution of microplastics (e.g. [5]), interactions between plastics and marine fauna are common [6]. Filter-feeding megafauna might be particularly affected by this form of pollution as their feeding mode requires filtration of hundreds to thousands of cubic meters of water [7].

The endangered whale shark (*Rhincodon typus* [8]) can spend 7.5 h day^− 1^ feeding in surface waters (0–1 m), and may thereby filter about 326 m^3^ of seawater h^− 1^, as estimated for a 4.4 m animal [9]. Even though the average mesh diameter of the filter pads may theoretically be permissive to smaller microplastics (i.e. < 1.2 mm [9]), whale sharks are also able to capture smaller particles, such as fish eggs (0.75–0.78 mm [10]), possibly through cross-flow filtration [9], thus being likely to reliably co-capture microplastics. Moreover, isotopic analyses have revealed that whale shark diets can consist of a diverse range of prey, such as shrimp and copepods, as well as myctophid fishes [11]–prey which has been reported to ingest microplastic in the natural environment [12–14]. Whale sharks thus not only ingest microplastics directly from the water, but further uptake could occur via trophic transfer [15]. Opportunistic sampling of stranded whale sharks has confirmed that indeed larger plastic items are ingested [16–19], but to date, evidence of direct microplastic ingestion is lacking.

The habitat range of whale sharks overlaps with several established microplastic pollution hotspots, such as the Coral Triangle [7, 20]. For the Coral Triangle, models have estimated that microplastics occur at 10^4^ to 10^6^ particles per km^2^ or 10^3^ g km^− 2^ [21]. This plastic pollution is in part sustained by inputs from several major rivers in the area, such as the Pasig and Mekong (midpoint input estimates ~ 10^4^ t yr^− 1^ [22]). Their contributions are likely to remain disproportionately high in the future, given projected volumes of mismanaged plastic waste [23]. Despite the high degree of plastic pollution in one of the world’s most biodiverse marine regions, surprisingly few studies on this topic have been conducted in the Philippines [24–26], and besides recently published data on microplastic concentrations in river mouths in the Manila bay area [27], no marine surface water microplastic concentrations have been reported.

Here, we investigate plastic ingestion non-invasively by analyzing scat samples from whale sharks feeding on surface waters in southeastern Cebu (Philippines). We aim to quantify plastic ingestion, characterize ingested plastics, and establish if temporal trends occur over a time span of 8 years, i.e. from 2012 to 2019. To obtain an estimate for local surface water microplastic pollution, we further collect and analyze water samples taken in 2019.

## Materials and methods

### Study site

This study was conducted in the municipality of Oslob, Cebu, Philippines. Even though this town is among the less densely populated towns on the island (ca. 28,000 compared to median 48,000; 2015 census), it generates comparatively high volumes of waste per capita (corresponding to median 0.31 kg day^− 1^) amounting to ca. 8563.15 kg of waste day^− 1^ (Cebu PENRO, 2017). The volume of waste per capita is high because it includes waste from tourism; Barangay (village) Tan-awan hosted the largest, provisioned, whale shark tourism site in the world receiving > 500,000 tourists yearly pre-SARS-CoV-2 pandemic [28]. From the town’s waste classification it is unclear however, what fraction of waste is represented by plastic: biodegradable (3767.79 kg day^− 1^), recyclable (1969.52 kg day^− 1^), residual (2740.21 kg day^− 1^), and special waste (85.63 kg day^− 1^). Plastic waste can be part of the latter three [29], although national data projects plastic waste to account for 11% of the total waste [30]. Nonetheless, waste management in Oslob is weak, as on the rest of the island of Cebu and for the entire country [29, 31]. The waste is still collected in unsegregated form and deposited in a local dumpsite [29]. There are no well-established mechanisms for composting nor recycling with an exception of few informal shops that purchase plastic waste (e.g. PET bottles) to be channeled for recycling locally or abroad [29, 31]. Since only coastal and urban areas are prioritized for waste collection, it is therefore not unusual that plastic waste leaks into the environment [29, 31].

### Whale shark scat samples

To assess microplastic ingestion in whale sharks, a total of 99 scat samples were analyzed, which had been collected from 2012 to 2019 by members of the Large Marine Vertebrates Research Institute (LAMAVE). The scat samples were opportunistically collected within the whale shark interaction area in Tan-awan during whale shark tourism hours (see [32]). Samples were hand-collected during in-water surveys and stored in polypropylene vials (6 or 10 mL; Perfector Scientific triple screw) filled with 98% Ethanol. For each sample were noted: whale shark identity through photo-identification, size and sex (following [32]), as well as sampling date and season. Samples were first dried in circular glass desiccators for 24 h, and then subsequently washed following an adapted method from Rebolledo et al. [33]. Each sample was transferred into a Teflon mesh-bag (6 cm × 4 cm, 240 μm mesh size), which was then closed with a sewing-machine and placed into a second Teflon mesh-bag (8 cm × 6 cm, 100 μm mesh size) and sewn shut. Samples were then washed in a laboratory washer (Hamo T-21) in two 70 °C washing cycles: one with enzymatic detergent (BIOTEX, Sara Lee H&HB Nederland B.V) containing subtilin, lipase, amylase and mannanase, and the second using regular commercially available clothes-washing detergent. After washing, the mesh-bags were opened and samples were filtered onto glass fiber filters (Rotilabo, Ø 90 mm, retention range 8–12 μm) and washed with Milli-Q water and ethanol 98% using a vacuum pump (Heidolph Rotavac valve control).

### Surface water samples

To determine local microplastic concentration in surface waters (0–1 m), a total of 3.6 m^− 3^ seawater were sampled off the southeast coast of Cebu. Sampling took place over 3 days in November 2019 at four sampling locations: two locations within the whale shark interaction area (9°27′45.1″N 123°22′52.3″E; 9°27′36.5″N 123°22′48.2″E), one location north of the interaction area (9°29′00.2″N 123°23′37.9″E), and one south of the interaction area (9°27′10.8″N 123°22′40.3″E). Each sample consisted of 0.9 m^− 3^ of surface water collected using a submersible pump (12 Volt Direct Current ELEGANT, Comet Pumps, Florida) at a depth of 1 m [34], meeting current sample size recommendations (≥ 0.5 m^− 3^) for analysis of microplastics > 300 μm [35]. The water was directly fed through three stacked steel sieves with mesh sizes of 5 mm, 1 mm and 0.3 mm. The material retained by the sieves was washed into previously rinsed glass jars (200 mL) using Milli-Q water. As biofouling was very limited and individual particles were visually distinguishable, samples were directly filtered onto glass fiber filters (Rotilabo, Ø 90 mm, retention range 8–12 μm) and stored for downstream analysis.

### Particle selection and identification of polymer type via ATR FTIR

All scat- and water sample filters were visually inspected for microplastics under a binocular microscope (45×, Leica Zoom 2000) and items ≥300 μm that fulfilled the following criteria were selected: (I) no visible cellular or organic structures, (II) fibers are equally thick throughout the entire length, (III) particles are homogeneously colored [36]. Then, all items were photographed under a binocular microscope (Olympus SZ61, 45× magnifying, camera: Olypmus SC50) and measured at their largest cross-section using Olympus CellSens software following Mani and Burkhardt-Holm [37]. MP particles were categorized according to their morphology and color as done previously (e.g. [38]).

To confirm the identity of suspected microplastics, attenuated total reflection Fourier-transform infrared spectroscopy (ATR FTIR) was used. Each visually-selected particle was placed manually on the crystal and compressed to record the spectrum in the range of 4000–400 cm^− 1^ with a resolution of 4 cm^− 1^ and over 24 scans (model Alpha, Bruker Optics GmbH). Spectra were then vector normalized (OPUS, version 7.5), and the first derivative was used for library searches in siMPle (https://simple-plastics.eu/) against a custom library for microplastic samples [39]. Assigned polymer types were accepted if the hit quality of the match was ≥70% [40]. Even though recorded, fibers and particles < 300 μm were excluded from further analyses due to technical constraints in determining polymer type, as items were too small for reliable handling and placing on the crystal to ascertain coverage.

### Quality control measures

Strict measures to limit contamination were taken. The laboratory workspace was wiped frequently and cleaned with a hand-held vacuum cleaner. For all steps where it was possible, analyses were performed in a horizontal flow hood (SKAN AG, Switzerland, model HFX.180BS), over which a cotton mosquito net was hung (for details see [38]). A white cotton laboratory coat was worn during all steps, including cotton clothing underneath, as well as blue vinyl gloves. All equipment used for analyses (i.e. tweezers, spoons, petri-dishes) was either steel or glass made and cleaned with 98% Ethanol between each sample. Before use, each glass fiber filter was visually inspected for contamination (i.e. fibers, particles or films).

To account for potential airborne contamination, blank glass fiber filters (Rotilabo, Ø 90 mm, retention range 8–12 μm) were moistened with Milli-Q water and placed in pre–cleaned petri dishes in direct vicinity to the working area and samples during all procedures, i.e., filling and sewing mesh-bags, washing process, filtration and visual inspection.

Finally, to detect potential contamination of water and scat samples previous to laboratory work, we extended our reference spectra library with spectra from the ship’s paint, and rope, as well as from the scat sample vials.

### Statistical analysis

To investigate whether microplastic concentrations in whale shark scat changed over time, a generalized linear model was used with a negative binomial distribution. The number of microplastics per sample weight (rounded to nearest integer) served as response variable, while sampling year and season were used as explanatory variables. Model fit was assessed through functions provided in the R package DHARMa [41], i.e. residual diagnostics and dispersion tests. Even though 19 of the 40 identified whale sharks were sampled more than once (repeats within and/or across years; Figure S1), each sample was treated as independent given the animal’s known use of the horizontal [42] and vertical [43] habitat at Oslob. Analyses and plotting were performed in R (version 4.0.3) [44] using the additional packages ggplot2 [45] and glmmTMB [46].

## Results

### Whale shark scat samples

**Fig. 1:**
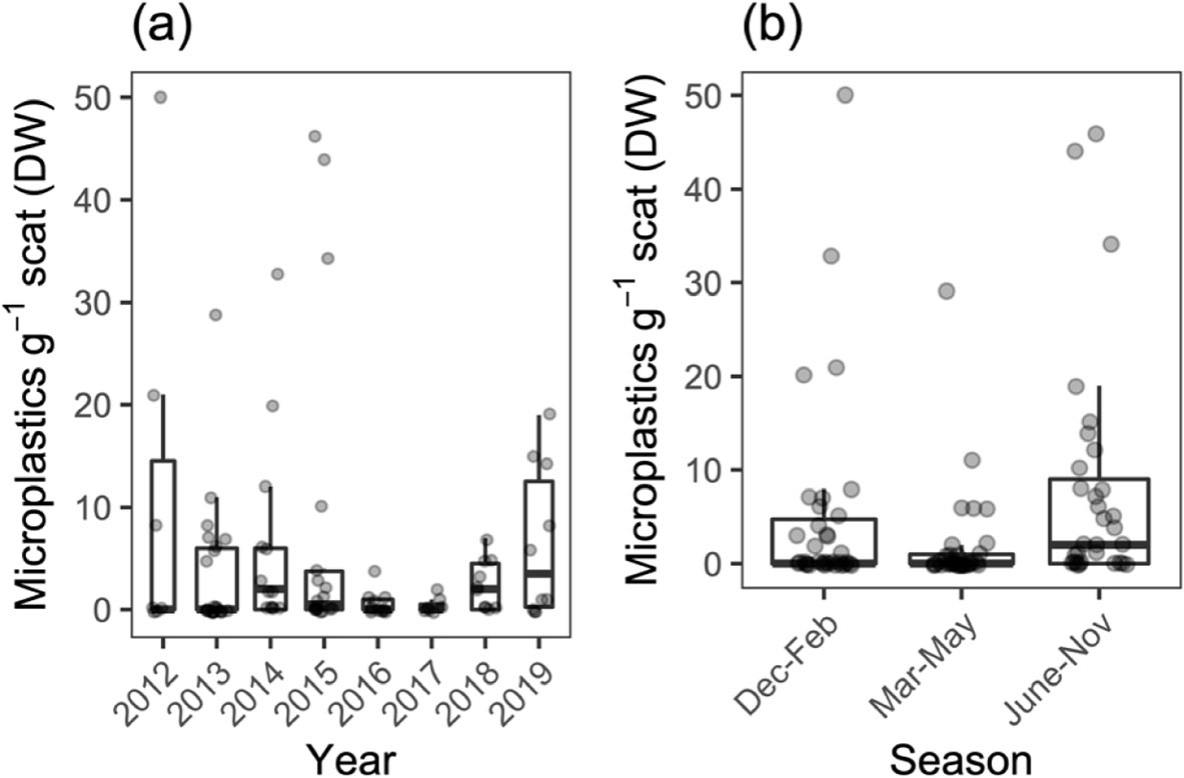
Number of microplastics per whale shark scat dry weight (DW) by **a** year and **b**season. Box plot width scaled to sample sizes (see Table 1)

Of the 99 scat samples, 89 stemmed from 40 identified individuals, of which 31 were male (mean size 4.7 m), and nine female (mean size 5.2 m). For the remaining 10 samples whale shark individuals could not be identified. The total scat dry weight was 63.78 g, with a per sample mean of 0.64 g (±0.55 S.D., Table 1). From the scat samples, we isolated 393 potential microplastic particles (MP, particle size > 300 μm due to technical constraints); FTIR analysis supported 46.5% (*n* = 179) of these to be of synthetic origin, and thus confirmed that 47 of the 99 samples contained at least 1 MP. We thus obtained 2.8 MP g^− 1^ scat, with individual samples spanning from 0 to 50 MP g^− 1^. Means ranged from 0.45 MP g^− 1^ in 2016 to 5.43 MP g^− 1^ in 2019 (Table 1, Fig. 1a). Our data of microplastic concentrations in scat do not provide support for increased incidence between 2012 and 2019 (GLM; year 2019, *p* = 0.27; Table S1), and samples from the intermediate years 2016 and 2017 had decreased microplastic concentrations relative to 2012 (GLM; year 2016, *p* = 0.005; year 2017, *p* = 0.032; Fig. 1a; Table S1). No support for a seasonal effect on concentrations of microplastics in whale shark scat was detected either (Fig. 1b, Table S1).

**Table 1:** Summary of whale shark fecal sample data

Year	n_samples_	Sample DW [g]^a^	Samples with MP [%]^b^	Total MP^c^	MP g^− 1^ sample	Particle size [mm]^d^	Samples by season^e^
2012	7	0.25 (±0.21)	42.9	7	5.71 (±9.43)	2.46 (1.63)	A: 7
2013	22	0.40 (±0.28)	31.8	23	1.82 (±3.29)	1.43 (0.80)	A: 5, B: 9, C: 8
2014	13	0.67 (±1.01)	61.5	23	3.23 (±5.09)	1.37 (0.93)	A: 7, B: 2, C: 4
2015	18	0.61 (±0.46)	55.6	46	4.06 (±7.82)	0.84 (0.40)	A: 6, B: 4, C: 8
2016	12	0.94 (±0.38)	33.3	5	0.25 (±0.62)	0.86 (0.17)	A: 4, B: 6, C: 2
2017	7	0.62 (±0.33)	28.6	2	0.29 (±0.49)	0.75 (0.27)	B: 6, C: 1
2018	10	0.70 (±0.28)	60.0	13	1.30 (±1.34)	1.07 (0.48)	A: 5, B: 4, C: 1
2019	10	1.11 (±0.58)	70.0	60	3.20 (±3.61)	1.02 (0.35)	B: 2, C: 8

^a^mean ± SD; *DW* Dry weight

^b^mean ± SD

^c^*MP* Microplastic particles

^d^mean ± SD; particle size corresponds to geometric mean

^e^A: December–February; B: March–May; C: June–November

**Fig. 2:**
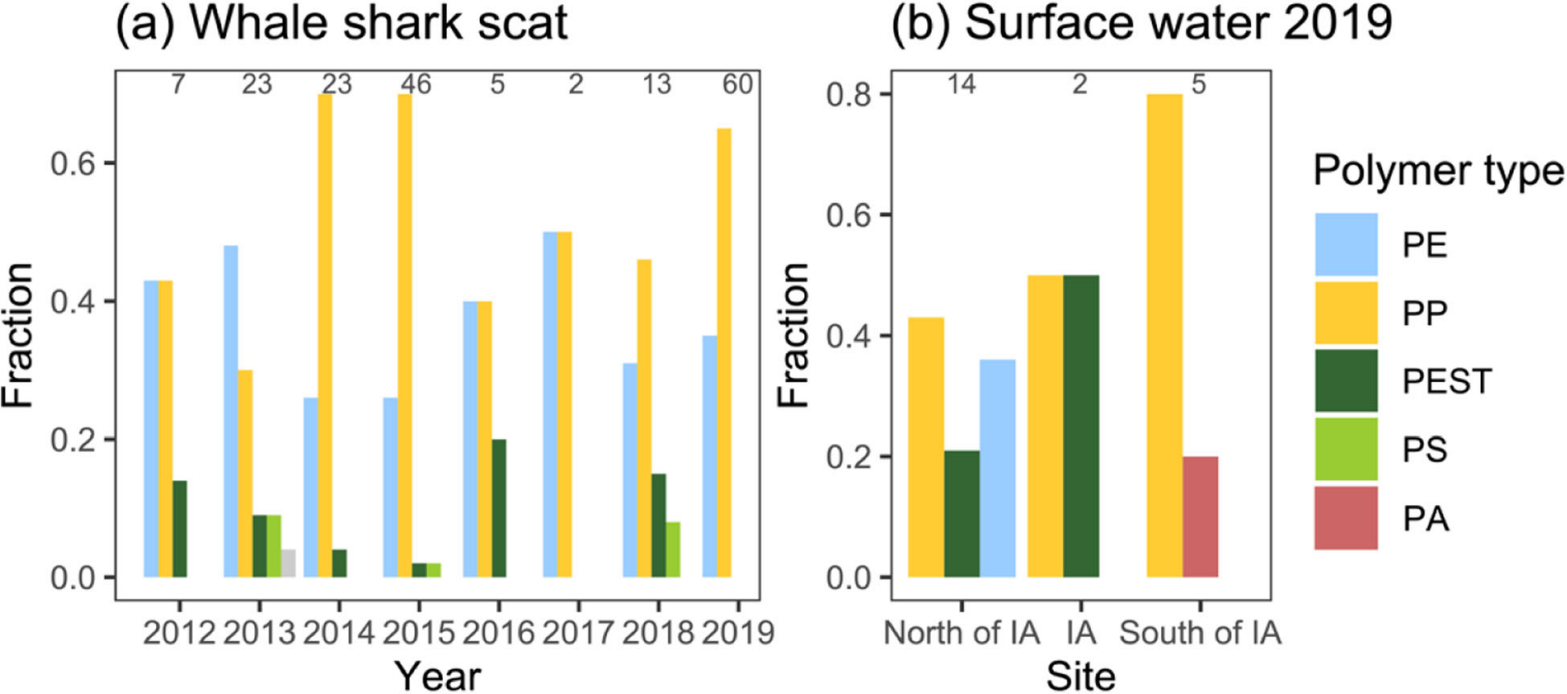
Fraction of microplastic polymer types in **a** whale shark scat samples across years and **b** surface water samples from sites around and in the whale shark interaction area (IA). PE: polyethylene; PP: polypropylene; PEST: polyester; PS: polystyrene; NR: nitrile rubber; PA: polyamide. Numbers above bars indicate microplastic counts for each **a** year or **b** site

Of the 179 identified microplastics, 173 were classified as fragments. The remaining six constituted fiber bundles, which were distinct from the fibers found in blank samples and thus included in the analysis (Figure S2; see quality control measures below). The overall mean particle size (±S.D.; geometric mean) was 1.12 (±0.7) mm, ranging from 0.84 mm in 2015 to 2.46 mm in 2012 (Table 1). The most abundant polymer type found in scat samples was polypropylene (PP) comprising 59.2% (*n* = 106) of the particles, followed by polyethylene (PE, 33.5%, *n* = 60), polyester (PEST, 4.5%, *n* = 8), polystyrene (PS, 2.2%, *n* = 4), and nitrile rubber (NR, 0.6%, n = 1). Some variation in relative polymer type abundance was observed between years, with PP appearing more dominant in 2014, 2015, and 2019 (Fig. 2). The majority of microplastics were transparent (54.8%, *n* = 98), blue (17.9%, *n* = 32) or white (14.5%, *n* = 26), while further identified colors comprised green (*n* = 7), yellow, grey (n = 6 each), and black, silver or orange (1 particle each). An additional 436 fibers of different colors (i.e. 288 blue, 47 transparent, 36 red, 28 green, 27 black, 7 yellow, and 3 purple) were recorded but excluded from analyses due to high contamination risk (see “Quality control measures” below) and uncertain FTIR confirmation.

### Surface water samples

From the overall 3.6 m^− 3^ of seawater sampled, we isolated 103 potential microplastics, of which 21 could be confirmed as microplastics, thus yielding a local microplastic concentration of 5.83 MP m^− 3^ of seawater. The lowest number of microplastics was found in the interaction area (2 in 1.8 m^− 3^), while the highest was obtained north of the interaction area (14 in 0.9 m^− 3^); and five microplastics were found in 0.9 m^− 3^ south of the interaction area.

Of the 21 microplastics from sea surface samples 17 were fragments, and four were fiber bundles. Particle sizes ranged from 0.16 to 1.57 mm (mean = 0.63 ± 0.34 mm). The relative abundance of polymer types was: PP (52.4%), PE (23.8%), PEST (19.0%), and polyamide (PA, 4.8%). Of the 17 fragments, six were transparent, five were grey, four were white, and one each were black and beige particles. Four fiber bundles were measured and included in the analysis (Figure S2). Additionally, 1055 differently colored fibers were isolated from water samples, i.e. 613 blue, 254 black, 87 red, 87 transparent, 9 green, 3 orange, and 2 yellow; these fibers were equally excluded from further as described above.

### Quality control measures

Analysis of the blank sample filters did not yield fragments nor films. Despite all efforts to prevent aerial contamination, however, we found a mean of 1 fiber 8 h^− 1^ of exposure. These fibers were predominantly blue (96%) and of unknown polymeric origin and thus excluded from the analysis. Exceptions were made for fiber bundles, which were recovered from scat and surface water samples, but never detected in our blank samples, and for which it was possible to assess the polymer type.

Most PP particles recovered from the scat samples were visually distinct from the material of the PP storage vials, as well as thinner and more bendable. Spectra library searches did not indicate the PP vials as likeliest source of any of the particles either.

## Discussion

We present a thorough assessment of whale shark scat for the first direct evidence of microplastic (MP) ingestion in this species. We used extensive longitudinal samples spanning 8 years (i.e. 2012–2019) and a representative number of individually identified whale sharks (*n* = 40). Microplastics were present in samples from all years (2.8 MP g^− 1^ scat), indicating the pervasiveness of microplastic ingestion amongst the world’s largest filter feeding fish in the largest, provisioned, whale shark tourism site at Oslob (Philippines). The most common polymers in scat samples were polypropylene PP (PP, 59.2%) and polyethylene (PE, 33.5%), which corresponds to the relative abundance found in surface waters and expected from filter-feeding in surface waters.

Direct evidence for microplastic ingestion in filter feeding megafauna remains sparse. A previous assessment of whale shark exposure to microplastic pollution relied on indirect measurements of persistent organic pollutants (POPs) in skin biopsy samples [47]. Similar studies have been conducted for other filter feeding megafauna, such as fin whales and basking sharks [48, 49]. It is, however, unclear to what extent the contamination of animal tissues with POPs can indeed serve as a proxy for microplastic ingestion. It should, for instance, be considered that microbial biofilms on microplastics degrade compounds such as bis(2-ethyl hexyl) phthalate (DEHP [50]), likely reducing transferred amounts. Even so, no evidence for microplastics serving as vectors for POPs was found in Northern Fulmars (*Fulmarus glacialis*, [51]). Indeed, the current consensus is that at present pollution levels microplastics play a minor role in transferring contaminants in comparison to other sources (e.g. directly from water or from suspended organic particles [52, 53]), highlighting the importance of obtaining direct evidence of microplastic ingestion in marine megafauna.

Microplastic pollution in surface waters within–and surrounding–the whale shark feeding area (5.83 MP m^− 3^) in November 2019 was one to two orders of magnitude higher than what is commonly found in surface waters elsewhere in the world [54], comparable to some more highly polluted areas, such as the Mediterranean sea (3.13 MP m^− 3^ [55]), or the Rhine river (5.6 MP m^− 3^ [56]). These findings represent the first data on microplastic concentrations in marine surface waters from the Philippines, apart from a preliminary assessment conducted in the Tañon Strait (0–1500 MP m^− 3^, total sampled V = 38 L [57]). The whale sharks feeding in the interaction area in Tan-awan thus appear more strongly exposed to microplastic pollution than in Java (Indonesia; 0.42 MP m^− 3^ [58]), but do not seem to be exposed to microplastic concentrations as high as that found in more polluted coastal Asian waters, such as estuaries in the East China Sea (100–4100 MP m^− 3^ [59]) or near Surabaya (490 MP m^− 3^ Indonesia [60]). The dominance of low-density-type polymers (i.e. PP and PE, 76.2%) in our surface water samples from 2019 is further congruent with previous results from surface water studies worldwide [54]. The similarly high percentage of PP and PE in the whale shark scat samples analyzed here from 2019, but also observed in preceding years (i.e. 2012–2018; Fig. 2) may thus reflect the filter feeding behavior of the assessed animals in surface waters. Assuming a water filtration rate of 326 m^3^ hr.^− 1^ for a 4.4 m whale shark [9], comparable to the mean size of the individuals sampled here (4.8 m), and a mean 7.5 h d^− 1^ spent feeding in surface waters [9], whale sharks surface feeding in Cebu in 2019 may theoretically have been ingesting ~ 14,000 MP day^− 1^, excluding microplastics from potentially contaminated prey. Compared to similar theoretical approximations from other whale shark feeding grounds, for Cebu we thus speculate that in 2019 whale sharks ingested 14× more microplastics than what was estimated for Java (1028 items day^− 1^ [58]) or 83× more than within La Paz Bay, Sea of Cortez (171 day^− 1^ [47]).

Despite finding microplastics in the scat samples, it is difficult to corroborate estimated microplastic ingestion rates based on water pollution levels and animal feeding behavior for several reasons. For instance, it is unknown what fraction of the total dry weight of an egestion event that our samples constituted. The uncertainty arises from the time point at which the sample can be caught, as whale shark scat is composed of fine sediment, which quickly disintegrates in water. If caught immediately after egestion, such scat can frequently weigh > 500 g, while only fractions can be obtained if caught deeper in the water column (GA, pers. obs.). Our samples with a mean weight of 0.64 g might only comprise ca. 0.13% of the full scat. Yet, with a mean of 2.8 MP g^− 1^ of scat found in our samples, the total number of egested microplastics per complete scat could indeed have comprised hundreds. In addition, the origin of the microplastics cannot be limited to water pollution. Due to the provisioning of whale sharks at the interaction area with previously processed food, egested microplastics may thus in part also stem from the feed. As provisioning in Oslob has also been observed to affect whale shark behavior, leading to a ca. six times increased period spent at < 2 m depth, compared to days without provisioning [43], a high degree of uncertainty remains. Moreover, it is unclear whether all macro- and microplastics are egested. Even though our opportunistic sampling did not allow to consider macroplastics, previous stomach content analyses from stranded individuals highlighted, that whale sharks ingest macroplastics, such as drinking straws and sheet-like items [16, 17, 19]. While Abreo et al. [16] did not find any indication of potential problems caused by the ingested plastics, Haetrakul et al. [17] reported stomach lacerations, and Matsumoto et al. [19] linked a macroplastic to the obstruction of pylorus and eventual death of that whale shark.

The impacts that microplastic ingestion may have on whale sharks remains difficult to assess. Experimental data obtained from studies on fish indicate that out of a total of 782 studied endpoints, 32% of them were significantly affected by microplastic exposure, whereby behavioral, sensory and neuromuscular endpoints were most often impacted (57% *n* = 100), followed by endpoints in the metabolism (34% on *n* = 305), and of the alimentary and excretory system (33% of *n* = 72 [61]). Although important, these results might not be directly transferable to animals in nature, as microplastic concentrations employed in laboratory studies commonly exceed the highest microplastic concentrations found in the natural world [62]. Further, control conditions frequently consist of particle free water, rather than including a natural form of debris [63], which fish are known to ingest [64, 65], and which can trigger similar cellular stress responses as reported for microplastics (e.g. oxidative stress [66]). Nonetheless, microplastic ingestion in surface waters has recently been found to correlate with modelled environmental microplastic abundance and to differ with ecological and behavioral traits of fish [67]. Even though filter-feeding fish appear to consume plastic less commonly than for instance active predators [67], if projected increases in marine plastic waste are realized, whale sharks will likely be subject to growing encounters with–and therefore ingestion of–microplastics, underscoring the importance to further elucidate any negative consequences of microplastic ingestion.

## Conclusions

Our study fills several research gaps by providing the first direct evidence of microplastic ingestion in whale sharks (mean of 2.8 microplastics g^− 1^ scat), as well as data on microplastic concentrations in coastal waters of southeastern Cebu, Philippines (5.83 particles m^− 3^). Even though microplastic pollution is below pollution hot-spots measured in South East Asia and China, whale sharks at the studied site are estimated to ingest 14,000 microplastics day^− 1^, based on typical surface feeding behavior. To what extent microplastic ingestion is impacting the overall health status of this endangered species, remains an open question. For better estimates of microplastic ingestion rates, better data on local whale shark feeding behavior and residency times should be obtained. Further investigation into linkages between microplastics readily available in the water column, and any assimilation by the animal’s tissues, should be prioritized, especially in regions where plastic pollution is pervasive, such as Southeast Asia. This may be achieved through activating stranding networks to collect and analyze gastrointestinal and liver tissues.

## Supplementary Information

**Additional file 1: Figure S1.** Number of scat samples collected from different photo identified whale sharks between 2012 and 2019. **Table S1.** Estimated microplastic incidence, confidence intervals (CI) and levels of significance (*p*), for generalized linear model of microplastics per sample weight in response to year and season. **Figure S2.** Examples of fiber bundles recovered from whale shark scat samples (a, b) and surface water samples (c, d). Lines and numbers indicate size measurements with corresponding values.

**Additional file 2.**

**Additional file 3.**

## Data Availability

All data used for this study are provided as supplementary information (“Data_WaterSamples.csv”; “Data_WhaleSharkScat.csv”).

## Abbreviations

MP: Microplastics
ATR FTIR: Attenuated total reflection Fourier-transform infrared spectroscopy
LAMAVE: Large Marine Vertebrates Research Institute
S.D.: Standard deviation
PP: Polypropylene
PE: Polyethylene
PEST: Polyester
PS: Polystyrene
NR: Nitrile rubber
PA: Polyamide
POP: Persistent organic pollutant
DEHP: Bis(2-ethyl hexyl) phthalate
