# Supplementary material for: Microplastics in fecal samples of whale sharks (Rhincodon typus) and from surface water in the Philippines
Source: Microplast nanoplast. 2021 Sep 26;1(1):17. doi: 10.1186/s43591-021-00017-9 (PMC8475362; doi:10.1186/s43591-021-00017-9)
Supplement: Supplementary file 1 — Additional file 1: Figure S1. Number of scat samples collected from different photo identified whale sharks between 2012 and 2019. Table S1. Estimated microplastic incidence, confidence intervals (CI) and levels of significance (p), for generalized linear model of microplastics per sample weight in response to year and season. Figure S2. Examples of fiber bundles recovered from whale shark scat samples (a, b) and surface water samples (c, d). Lines and numbers indicate size measurements with corresponding values. [file 43591_2021_17_MOESM1_ESM.pdf]

SUPPLEMENTARY INFORMATION

Microplastics in fecal samples of whale sharks (*Rhincodon typus*) and from surface water in the Philippines

Mila Yong[1], Clara Leistenschneider[1], Joni Anne Miranda[2], Maria Kristina Paler[3], Christine Legaspi[2], Elitza Germanov[4], Gonzalo Araujo[2,5], Patricia Burkhardt-Holm[1], Gabriel Erni-Cassola[1]

[1]Program Man-Society-Environment, University of Basel, Vesalgasse 1, CH-4051 Basel

[2]Large Marine Vertebrates Research Institute Philippines, Jagna, Bohol, 6308, Philippines

[3]Department of Biology, University of San Carlos, Talamban, Cebu City, Philippines

[4]Marine Megafauna Foundation, 11260 Donner Pass Road, #256 Truckee, CA 96161 USA

[5]Marine Research and Conservation Foundation, Somerset, TA4 3SJ, UK

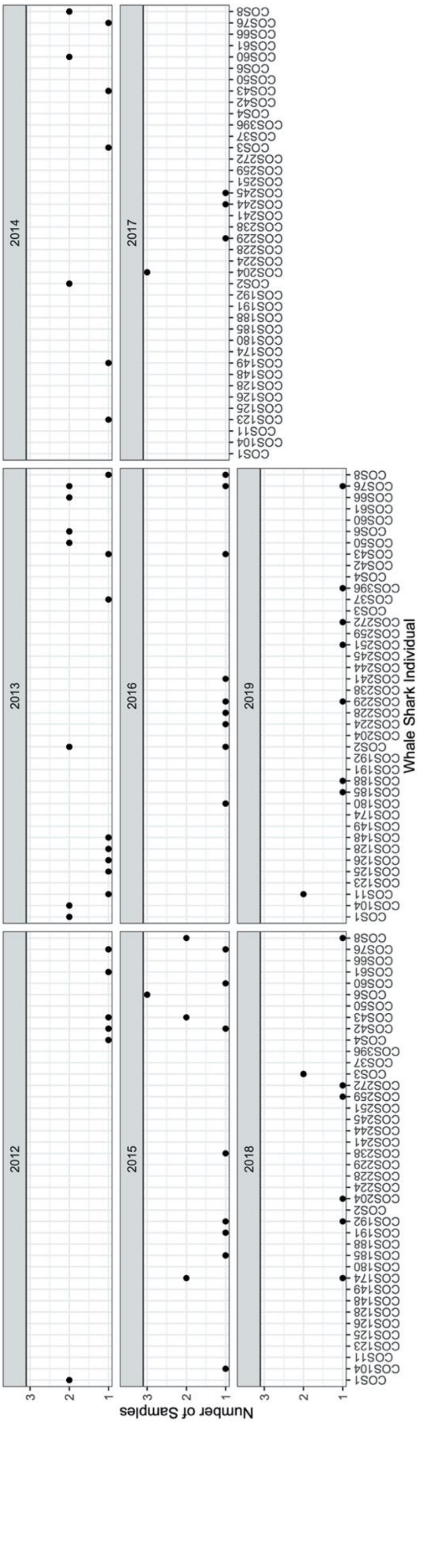

Figure S1. Number of scat samples collected from different photo identified whale sharks between 2012 and 2019.

Table S1. Estimated microplastic incidence, confidence intervals (CI) and levels of significance (*p*), for generalized linear model of microplastics per sample weight in response to year and season.

**Predictors**	**Incidence Rate Ratios**	**CI**	***p***
Intercept (Year [2012], Season [A][a])	11.29	2.67 – 47.74	0.001
Year [2013]	0.35	0.05 – 2.47	0.291
Year [2014]	0.54	0.09 – 3.39	0.514
Year [2015]	0.33	0.04 – 3.08	0.330
Year [2016]	0.05	0.01 – 0.42	0.005
Year [2017]	0.05	0.00 – 0.78	0.032
Year [2018]	0.20	0.03 – 1.38	0.102
Year [2019]	0.26	0.02 – 2.92	0.274
Season [B] [a]	0.69	0.19 – 2.52	0.576
Season [C] [a]	2.30	0.47 – 11.27	0.303
Observations	99		

[a]A: December–February; B: March–May; C: June–November

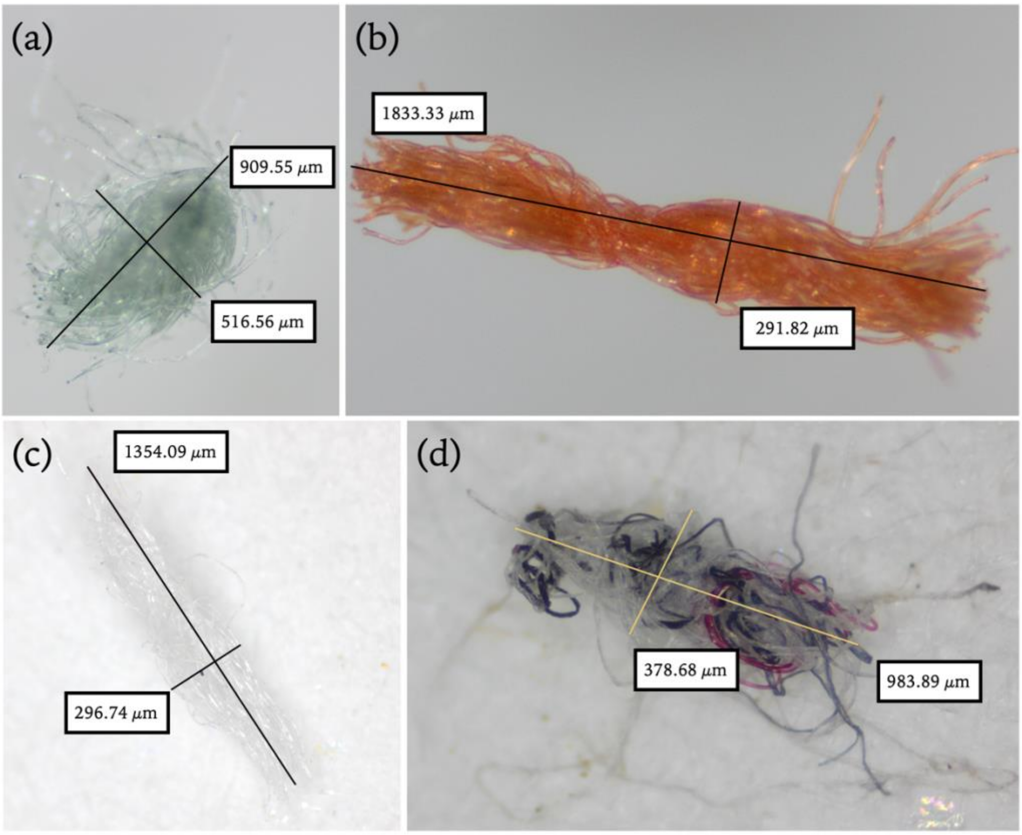

Figure S2. Examples of fiber bundles recovered from whale shark scat samples (a, b) and surface water samples (c, d). Lines and numbers indicate size measurements with corresponding values.
